# The importance of antioxidant status in gastric intestinal metaplasia

**DOI:** 10.5937/jomb0-29349

**Published:** 2021-09-03

**Authors:** Nilay Danis, Aysegul Ertinmaz Ozkan, Fatih Karatas, Cagri Cakici, Turkan Yigitbasi, Nurhayat Ozkan Sevencan, Burcak Kayhan

**Affiliations:** 1 Karabuk University Education and Research Hospital, Gastroenterology, Karabuk, Turkey; 2 Karabuk University Education and Research Hospital, Department of Internal Madicine, Karabuk, Turkey; 3 Karabuk University Education and Research Hospital, Medical Oncology, Karabuk, Turkey; 4 Medipol University, Regenerative and Restorative Medicine Research Center, Istanbul, Turkey

**Keywords:** intestinal metaplasia, oxidative stress, tiol, disulphide, intestinalna metaplazija, oksidativni stres, tiol, disulfid

## Abstract

**Background:**

Oxidative stress status in different cancer types was investigated before, but not studied in gastric intestinal metaplasia to the best of our knowledge. Purpose of this study is to examine whether there is a difference between oxidative stress status in patients with intestinal metaplasia (IM) compared to individuals without IM, we compared the serum levels of disulfide (SS), total thiol (TT) and native thiol (NT).

**Methods:**

This was a prospective, non-randomized casecontrol study including 67 patients with histopathologically confirmed IM and 60 individuals demographically matched in terms of age, gender, BMI, smoking status, and chronic diseases as control group.

**Results:**

The mean NT, TT and NT to TT (NT/TT) ratios were statistically significantly higher in IM group compared to controls ((351.71 ± 81.9 mol/L vs. 271.82 ± 54.13 mol/L, p=0.000), (391.5 ± 92.69 mol/L vs. 308.59 ± 55.53 mol/L, p=0.000) and (0.89 ± 0.6 vs. 0.87 ± 0.29, p=0.022), respectively). The mean SS to TT (SS/TT) ratio was significantly lower in IM group than control group (0.050 ± 0.31 vs. 0.060 ± 0.014, P=0.022). Median SS and mean SS/NT ratio was similar in both groups (16.3 (3.3-78) vs. 18.3 (10-32.7), p=0.271 and 0.055 ± 0.041 vs. 0.070 ± 0.019, p=0.068, respectively). In ROC analysis, cut off value of SS/NT for IM was found 0.062, in regression analysis, SS/NT <0.062 was found as an independently prognostic marker for IM (OR, 2.38; 95%CI: 1.168-4.865, P=0.017).

**Conclusions:**

SS/NT ratio lower than 0.062 was found as an independently prognostic marker for IM. This ratio could help to distinguish which patients should be followed closely for gastric cancer.

## Abstract (sr)

**Uvod:**

Status oksidativnog stresa kod različitih vrsta karcinoma istražen je ranije, ali nije proučavan u intestinalnoj metaplaziji želuca prema našem najboljem znanju. Svrha ove studije je da ispita da li postoji razlika između statusa oksidativnog stresa kod pacijenata sa intestinalnom metaplazijom (IM) u poređenju sa osobama bez IM, tako što su upoređeni nivoi serumskog disulfida (SS), ukupnog tiola (TT) i nativnog tiola (NT).

**Metode:**

Ovo je bila prospektivna, nerandomizirana studija slučaja i kontrole, koja je obuhvatala 67 pacijenata sa histo patološki potvrđenim IM i 60 osoba demografski podudarnih u pogledu starosti, pola, BMI-a, statusa pušenja i hroničnih bolesti kao kontrolna grupa.

**Rezultati:**

Srednji odnosi NT, TT i NT prema TT (NT/TT) bili su statistički značajno veći u IM grupi u poređenju sa kontrolama (351,71 ± 81,9 mol/L naspram 271,82 ± 54,13 mol/L, p=0,000), (391,5 ± 92,69 mol/L na - suprot 308,59 ± 55,53 mol/L, p=0,000) i (0,89 ± 0,6 naspram 0,87 ± 0,29, p=0,022), respektivno. Srednji odnos SS prema TT (SS/TT) bio je značajno niži u IM grupi od kontrolne grupe (0,050 ± 0,31 naspram 0,060 ± 0,014, P=0,022). Medijana SS i srednji odnos SS/NT bili su slični u obe grupe 16,3 (3,3-78) naspram 18,3 (10-32,7), p=0,271 i 0,055 ± 0,041 naspram 0,070 ± 0,019, p=0,068, respektivno). U ROC analizi utvrđena je granična vrednost SS/NT za IM 0,062, u regresionoj analizi SS/NT <0,062 kao nezavisno prognostički marker za IM (OR, 2,38; 95% CI: 1,168-4,865, P=0,017).

**Zaključak:**

SS/NT odnos niži od 0,062 pronađen je kao nezavisno prognostički marker za IM. Ovaj odnos bi mogao da pomogne da se dijagnostikuju pacijenti kod kojih treba pažljivo pratiti rak želuca.

## Introduction

Gastric cancer is the 5^th^ most common neoplasm, and it is the 3^rd^ mortal cancer [1]. Early gastric cancer prognosis is relatively better, however, it is generally asymptomatic. Unfortunately, 65-80% of gastric cancers is diagnosed in advanced stage, this directly influences overall survival [2]. More than 90% of gastric cancer is adenocarcinoma histopathologically, and adenocarcinoma mainly involves two subtypes; a) well-differentiated or intestinal type, b) poorly differentiated or diffuse type [2]. Pre-malign gastric lesions are well-defined lesions for intestinal type gastric adenocarcinogenesis. These lesions cascade proceeds chronic inflammation due to *helicobacter pylori* infection, atrophic gastritis, intestinal metaplasia (IM), dysplasia, and cancer [3]. Intestinal metaplasia is the transformation (metaplasia) of epithelium, usually of the stomach or the esophagus, to a type that bears some resemblance to the intestine. Chronic infection caused by *helicobacter pylori* infection in the stomach is seen as the primary trigger of metaplasia and subsequent adenocarcinoma formation. Initially, the transformed epithelium resembles to the small intestine; in the later stages, the epithelium resembles the colon. It is characterized by the appearance of goblet cells and expression of intestinal cell markers such as Cdx2. Identified risk factors for IM include *helicobacter pylori* infection, high salt intake, smoking, alcohol consumption, and chronic bile reflux [4]. *Helicobacter pylori *is very common in our country, and its prevalence is 82.5% according to TURHEP study [5]. It could be asserted that this cascade could be the most active pathway in intestinal type gastric adenocarcinoma in our country. As gastric cancer's only curative method is still surgery, it creates a big difference for prognosis to diagnose cancer in early stages. It seems wise to follow-up a case with IM to detect early stage gastric cancer [6]. Since endoscopy is an invasive method, investigators have been in search of prognostic biomarkers that can be obtained easily, and inexpensively. It has been accepted that oxidative stress plays a pivotal role for tumor priming, progression and metastasis [7].

Reactive oxygen species (ROS) are physiologically produced by aerobic cells and this production increases in case of cellular damage. Physiological levels of ROS mediate critical intracellular survival signaling pathways. In addition, ROS itself, which is present in high levels of inflamed tissue, can also cause an inflammatory process [8]. The cells increased inflammatory process can activate multiple inflammatory and proliferative pathways at the cellular level, thus ROS seem to have great importance in tumorigenesis. In addition, oxidative stress causes a cellular redox imbalance, which leads to disruptions in apoptoticanti-tumorogenesis pathways. This redox imbalance has been found in various cancer, including lung, prostate cancer [9] [10] [11] [12] [13]. Oxidative stress is broken by the antioxidant molecules in the environment. Oxidative stress increases excessively as a result of the deficiency, failure or depletion of molecules that provide an antioxidant-redox balance or because of ROS molecules. The effect of ROS is balanced with enzymatic and non-enzymatic antioxidants (e.g. superoxide dismutase (SOD), catalase (CAT) and glutathione peroxidase (GPX) [9]. Thiol is an organic compound containing a sulfhydryl (-SH) group, which plays a critical role in preventing the occurrence of any oxidative stress in cells. Thiol groups of sulfur-containing amino acids (cysteine, methionine etc.) in proteins are the primary target point of ROS. Together with ROS, thiol groups in the environment are oxidized and turned into reversible disulfide bonds. This transformation is the earliest sign of radical-mediated protein oxidation. Dynamic thiol/disulfide balance status has critical roles in antioxidant defense, detoxification, apoptosis, regulation of enzyme activities, transcription and cellular signal transduction mechanisms [14]. Previous studies have found that thiol/disulfide balance levels are involved in cancer pathogenesis and are associated with prostate cancer, lung, endometrium cancer, ovarian cancer, and gastric cancer [9] [11] [13] [14] [15] [16]. According to our knowledge, thiol levels in IM were not investigated so far. The aim of this study is to detect the difference of thiol disulfide homeostasis markers such as serum native thiol (NT), total thiol (TT) and disulfide (SS) levels between control subjects and in previously histologically diagnosed patients with intestinal metaplasia, and to present possible new evidence with the mechanism of the disease.

## Material and Methods

**Figure 1:**
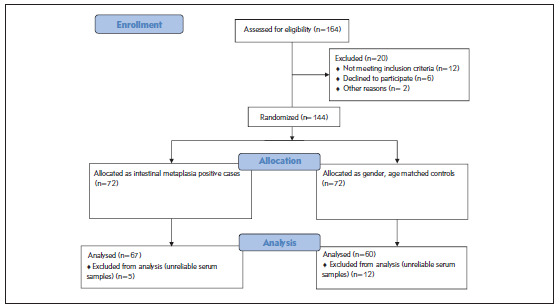
Consort flow diagram of patients

This was a prospective, non-randomized, and case-control study with a total of 127 subjects, including 67 patients with histopathologically confirmed IM and 60 controls who were demographically matched in terms of age, gender, BMI, smoking status, and chronic diseases (i.e. Diabetes Mellitus, Hypertension, and Coronary Artery Disease) (Figure 1).

Intestinal metaplasia diagnosis was confirmed by discussing pathology specialists, and IM of cardia was excluded. Only patients with distal gastric IM were included into the study. Other exclusion criteria were subjects younger than 18 years old, gastric operation history, any malignancy history, pregnancy, lactating mothers, smokers, having organ dysfunction such as liver, kidney, and thyroid, patients unable to feed orally, any presence of rheumatic disorder, use of anti-inflammatory or immunosuppressive drugs (i.e. non-steroidal anti-inflammatory drugs, corticosteroids, anti-tumor necrosis factor alpha, or colchicine), and receiving hormone replacement therapies.

### Laboratory work-up

At least 12 hours fasting peripheral venous blood samples were collected from both patients and controls at in morning at 8:00 am. These samples were stored for 20 minutes for clothing. Then, they were centrifuged for about 10 min at 1500 g. The separated serums were stored immediately for equal periods in the freezer at -80°C (Median 12 [1] [2] [3] [4] [5] [6] [7] [8] [9] [10] [11] [12] [13] [14] [15] [16] weeks) for both controls and IM patients. Levels of TT, NT, and SS were measured through a recent automatic and spectrophotometric method. Initially, we separated free thiol groups by reducing the disulfide bonds using sodium borohydride. Formaldehyde was used to suppress the unused part of 5,50-dithiobis-2nitrobenzoic acid (DTNB), with the goal of preventing its reduction, then, TT groups containing reduced and NT groups were identified. Dynamic SS was calculated through the method of dividing the alteration between TT and NT. The rates of SS/TT, SS/NT, and NT/TT were numbered as per their percentages [13].

### Statistical analysis

Statistical analyses were performed via Windows SPSS program (version 22.0, SPSS Inc., Chicago, IL). Kolmogorov-Smirnov and the Shapiro-Wilk tests were used to analyze whether the data were normally distributed. For non-normally distributed data, median and min-max values were analyzed, whereas mean and standard deviation were analyzed for normally distributed data. In order to compare the normally distributed data, Student t-test was used, whereas Mann-Whitney U test was used for the comparison of non-normally distributed data. Chi-square or Fisher exact test was utilized to assess the categorical variables. Post hoc analysis was performed to identify the differences between the groups.

### Ethical approval

As stated in the 1964 Helsinki declaration and its subsequent amendments, all of the methods including human subscribers in our study were carried out in compliance with the ethical standards of national research committee. Written informed consent was taken from all participants included in this study, after obtaining ethics committee approval the clinical trials ethics committee of Karabuk University Faculty of Medicine, a tertiary level health care center, approved this study with the decision number 3/41 in March 2019.

## Results

In our study, there were 127 subjects, including 67 patients with IM and 60 controls. Demographic data of all subjects were summarized in (Table 1).

**Table 1:** Main characteristics of all groups (n=127) SD, standard deviation.

Variables		All group	
Age (years), mean ± SD		54.86 ± 11.22	
Sex, n (%) Female Male		58 (45.7) 69 (54.3)	
Native thiol (µmol/L), mean ± SD		313.96 ± 80.5	
Total thiol (µmol/L), mean ± SD		352.33 ± 87.58	
Disulphyde (µmol/L), median ± SD		17.53 (3.3–78)	
Disulfide/total thiol ratio, mean ± SD		0.055 ± 0.25	
Disulfide/native thiol ratio, mean ± SD		0.064 ± 0.033	
Native thiol/total thiol ratio, mean ± SD		0.88 ± 0.5	

NT, TT and NT/TT ratios were statistically significantly higher in IM group compared to controls ((351.71 ± 81.9 µmol/L vs. 271.82 ± 54.13 µmol/L, p=0.000), (391.5 ± 92.69 µmol/L vs. 308.59 ± 55.53 µmol/L, p=0.000) and (0.89 ± 0.6 vs. 0.87 ± 0.29, p=0.022), respectively), whereas SS/TT ratio was statistically significantly lower in IM group than control subjects (0.050 ± 0.31 vs. 0.060 ± 0.014, P=0.022). SS and SS/NT ratio was similar in both groups (16.3 (3.3–78) vs. 18.3 (10–32.7), P=0.271and 0.055 ± 0.041 vs. 0.070 ± 0.019, P=0.068, respectively). Comparison of two groups was summarized in (Table 2).

**Table 2:** Comparison of demographic data and thiol levels between subjects with intestinal metaplasia and controls (n=127) SD, standard deviation.

Variables	Intestinal metaplasia (n=67)	Control group (n=60)	p
Age (years), mean ± SD	56.07 ± 13.16	53.51 ± 8.46	0.201
Sex, n (%) Female Male	33 (49.3) 34 (50.7)	25 (41.7) 35 (58.3)	0.476
Native thiol (µmol/L), mean ± SD	351.71 ± 81.9	271.82 ± 54.13	0.000
Total thiol (µmol/L), mean ± SD	391.5 ± 92.69	308.59 ± 55.53	0.000
Disulphyde (µmol/L), median ± SD	16.3 (3.3 – 78)	18.3 (10 – 32.7)	0.271
Disulfide/total thiol ratio, mean ± SD	0.050 ± 0.31	0.060 ± 0.014	0.022
Disulfide/native thiol ratio, mean ± SD	0.055 ± 0.041	0.070 ± 0.019	0.068
Native thiol/total thiol ratio, mean ± SD	0.89 ± 0.6	0.87 ± 0.29	0.024

In ROC analysis, NT cut-off value for IM was 300.6 µmol/L (77% sensitivity and 73 specifity, area: 0.790; 95%CI: 0.712-0.868; p= 0.001), TT cut-off value; 346.85 µmol/L (73% sensitivity and 73% specifity, area; 0.795, 95%CI: 0.718-0.872; p= 0.000), NT/TT cut-off value was; 0.88 (64% sensitivity, 63% specifity, area 0.674, 95%CI: 0.574-0.774, p=0.001), SS/SH cut-off value; 0.062 (62% sensitivity and 62% specifity, area 0.674, 95%CI: 0.574-0.773, P=0.001) and SS/TT cut of value; 0.055 (62% sensitivity and 62% specifity, area 0.675, 95%CI: 0.575–0.774, p=0.001)

There was no correlation between age and any of thiol parameters, but gender was correlated with thiol levels. Serum SS, SS/SH and SS/TT ratios were statistically significantly lower in women compared to men, whereas SH/Total thiol ratios were significantly higher in women (15.6 (3.9–46) vs. 18.5 (3.3–78), p=0.014, 0.057 ± 0.037 vs. 0.07 ± 0.33; p=0.036, 0.050 ± 0.22 vs. 0.06 ± 0.024; p=0.028 and 0.089 ± 0.051 vs. 0.087 ± 0.048; p=0.029).

In multivariate regression analysis, only SS/NT ratio lower than 0.062 was found as an independently prognostic marker for IM (OR, 2.38; 95%CI: 1.168-4.865, P=0.017) (Table 3).

**Table 3:** Evaluation, correlation, and multivariate logistic regression analysis results of factors contributing to intestinal metaplasia progression

Variables	OR	95% Confidence interval	P
Sex Female Male	1 1.18	0.312–1.89	0.563
Age	1.02	0.988–1.064	0.225
Disulfide/total thiol ratio <0.055 >0.055	1.317 1	0.187–9.225	0.782
Disulfide/native thiol ratio <0.062 >0.062	2.38 1	1.168–4.865	0.017
Native thiol/total thiol ratio <0.88 >0.88	1 2.42	0.471–12.523	0.289

## Discussion

According to the findings of our study, the levels of NT, TT and NT/TT ratios were statistically significantly higher in IM group compared to controls. We also found that SS/TT ratio was statistically significantly lower in IM group than control subjects. In our study, in logistic regression analysis, only SS/NT ratio lower than 0.062 was found as an independently prognostic marker for IM with an OD: 2.38. Dynamic thiol/SS balance is one of the most important parts of the antioxidant system in the human body. In case of incurring oxidative stress, tissues respond with antioxidant system. Native tiols in the antioxidant pool form disulfides after being reduced by reactive oxygen radicals. This amount of SS is actually an indirect measure of the body's response to oxidative stress. NTs in the environment need to decrease, while the amount of SS needs to increase, hence TT levels will ultimately increase to make a contribution to the antioxidant system. In our study, NTs, TTs, native/total thiols ratios were found to increase, on the other side disulfides/total thiol ratio were found to decrease, on the contrary. This means the lack of antioxidant system in IM similar to proliferative processes. A recently published study revealed that total antioxidant status was lower in patients with gastric cancer than controls, and unsurprisingly total oxidant status was greater in gastric cancer patients [16]. There is more evidence in the literature for increased radical oxygen species in the gastric cancer [17]. Another study determined severe oxidative stress in patients with gastric cancer, similarly but lower oxidative stress in patients with atrophic gastritis [18]. Our study demonstrated that antioxidant system was defective in patients with IM. Considering, the cascade of intestinal type gastric cancer patogenesis, it could be asserted that in the earlier phases of cascade, (atrophic gastritis) tissues encounter oxidative stress, but could not respond with enough antioxidative system (IM), finally in gastric cancer phases the oxidative stress increases.

In our study, there was a correlation with thiol levels and gender. We found that serum disulfide levels, SS/SH and SS/TT ratios were statistically significantly lower in women compared to men. It is well-known fact that gastric cancer is more prevalent among men compared to women, and gastric cancer is 2.2 times more likely to be diagnosed in males than females [19]. Differences serum thiol levels between genders could be one of the pathophysiological mechanisms underlying gastric cancers.

Strengths of our study are as follows: 1) according to our knowledge this is the first study investigating the antioxidant system in IM, although it was investigated in previous studies in gastric cancer [16] [17] [18]. Second, our control group were similar in terms of demographic and co morbid features; third, the blood samples taken from control and patient group were kept at similar durations, hence minimizing the factors that may affect the study in order to provide more refined data.

On the other hand; limitations of our study were we could not classify the IM group such as extensive or focal; incomplete and complete. It is known that these subtypes have different behavioral patterns, and surveillance protocol for gastric cancer [20].

In conclusion, it was firstly declared that there is a lack of antioxidant system in earlier phase of carcinoma. Disulphide/native thiol ratio lower than 0.062 was found as an independently prognostic marker for IM with an OR as 2.38. But more studies are needed to enlighten the effect of antioxidant / oxidant system role in the pathogenesis from chronic inflammations to advanced gastric cancer.

## Conflict of interest statement

All the authors declare that they have no conflict of interest in this work.
